# Editorial: Construction of regulatory networks in plant abiotic stress responses, volume II

**DOI:** 10.3389/fpls.2023.1195076

**Published:** 2023-04-12

**Authors:** Ji Huang, Henrik Aronsson, Danhua Jiang

**Affiliations:** ^1^ State Key Laboratory of Crop Genetics & Germplasm Enhancement and Utilization, Nanjing Agricultural University, Nanjing, China; ^2^ Department of Biological & Environmental Sciences, University of Gothenburg, Gothenburg, Sweden; ^3^ Institute of Genetics and Developmental Biology, Chinese Academy of Sciences, Beijing, China

**Keywords:** abiotic stress, regulatory network, non-coding RNA, alternative splicing, transcription factor

In recent years, extreme weather conditions occur more frequently as the global climate changes. As a consequence, abiotic stresses such as drought, high/low temperatures, salinity, and flooding are becoming more prominent, which adversely affecting plant growth, development and agricultural productivity. To cope with the challenge of abiotic stress, plants transmit the stress signals within cells and across cells and tissues *via* intricate molecular control, so as to regulate plant physiological development and enhance their adaptability. The construction and understanding of the abiotic stress regulation network will provide valuable genomic resources for molecular breeding of agriculture. In this special issue, Li et al. focused on the studies of regulatory mechanisms of non-coding RNAs (ncRNAs) in response to drought stress in *Triticum aestivum* (wheat). In this study, the long non-coding RNAs (lncRNAs), micro RNAs (miRNAs), and mRNAs related to drought stress were identified by transcriptome sequencing. On the combined analyses of these differentially expressed lncRNAs (DELs), differentially expressed miRNAs (DEMs), and differentially expressed genes (DEGs), 10 lncRNA-miRNA-mRNA regulatory modules related to wheat drought stress response were screened and two important candidate modules of them were verified by qRT-PCR. The results showed that ncRNAs play an important role in drought stress response by regulating gene expression. The prediction of regulatory patterns of these candidate lncRNA-miRNA-mRNA modules related to drought tolerance provided useful information in molecular breeding of wheat. Yang et al. also studied the differential expression of ncRNAs in response to drought stress in *Phyllostachys aureosulcata f. spectabilis* (bamboo). In this research, ncRNAs including circular RNAs (circRNAs) in addition to conventional lncRNAs and miRNAs were identified and analyzed. A total of 2,419 differentially expressed ncRNAs were detected, and the performed Gene Onthology (GO) and KEGG enrichment analysis showed that the host genes were enriched mainly in biochemical reactions involved in some metabolites, as well as in organelle activities. Thus, ncRNAs may regulate the metabolism of multiple metabolites to cope with drought stress. Understanding the regulatory mechanisms of ncRNAs in response to drought will provide a molecular basis for plant resistance studies. Fan et al. focused in a review paper on the research of links between circadian clock and alternative splicing (AS) of plant life activities responsive to abiotic stresses. They reviewed prior studies demonstrating that splicing factors could affect circadian rhythm by itself and the AS of core circadian genes under abiotic stresses. In turn, some genes could undergo AS according to circadian rhythm changes in abiotic stress responses. The crosstalk of circadian clock and AS under abiotic challenges in plants suggests a novel regulatory network that offers new ways to deal with abiotic stress. Leaf senescence is also a response to abiotic stress such as nitrogen deficiency. Wen et al. present the regulatory network of N deficiency-induced leaf senescence in *Malus domestica* (apple), and shows that overexpressing *MdNAC4* enhanced the senescence phenotype of leaves after N deficiency. Instead, overexpression of *MdAPRR2* stimulates the expression of chlorophyll synthesis-related genes, which can delay leaf senescence. It is suggested that there may be a balancing mechanism between MdNAC4 and MdAPRR2 in response to N deficiency through the interaction between MdNAC4 and MdAPRR2. It is necessary to summarize the existing research on the molecular mechanisms of plant stress response, especially to sort out the crosstalk among the different regulatory networks, and construct a systematic regulation network for plant abiotic stress responses. It will ultimately be applied to the breeding of highly resistant crops.

## Author contributions

JH drafted the manuscript. HA and DJ revised the manuscript. All authors approved the final version of manuscript.

